# RAviz: a visualization tool for detecting false-positive alignments in repetitive genomic regions

**DOI:** 10.1093/hr/uhac161

**Published:** 2022-08-01

**Authors:** Dong Xu, Yu Song, Xianjia Zhao, Desheng Gong, Yingxue Yang, Weihua Pan

**Affiliations:** Shenzhen Branch, Guangdong Laboratory of Lingnan Modern Agriculture, Genome Analysis Laboratory of the Ministry of Agriculture and Rural Affairs, Agricultural Genomics Institute at Shenzhen, Chinese Academy of Agricultural Sciences, Shenzhen 518120, China; Shenzhen Branch, Guangdong Laboratory of Lingnan Modern Agriculture, Genome Analysis Laboratory of the Ministry of Agriculture and Rural Affairs, Agricultural Genomics Institute at Shenzhen, Chinese Academy of Agricultural Sciences, Shenzhen 518120, China; Zhengzhou Research Base, State Key Laboratory of Cotton Biology, School of Agricultural Sciences, Zhengzhou University, Henan Zhengzhou 450001, China; Shenzhen Branch, Guangdong Laboratory of Lingnan Modern Agriculture, Genome Analysis Laboratory of the Ministry of Agriculture and Rural Affairs, Agricultural Genomics Institute at Shenzhen, Chinese Academy of Agricultural Sciences, Shenzhen 518120, China; Zhengzhou Research Base, State Key Laboratory of Cotton Biology, School of Agricultural Sciences, Zhengzhou University, Henan Zhengzhou 450001, China; Shenzhen Branch, Guangdong Laboratory of Lingnan Modern Agriculture, Genome Analysis Laboratory of the Ministry of Agriculture and Rural Affairs, Agricultural Genomics Institute at Shenzhen, Chinese Academy of Agricultural Sciences, Shenzhen 518120, China; Shenzhen Branch, Guangdong Laboratory of Lingnan Modern Agriculture, Genome Analysis Laboratory of the Ministry of Agriculture and Rural Affairs, Agricultural Genomics Institute at Shenzhen, Chinese Academy of Agricultural Sciences, Shenzhen 518120, China; Shenzhen Branch, Guangdong Laboratory of Lingnan Modern Agriculture, Genome Analysis Laboratory of the Ministry of Agriculture and Rural Affairs, Agricultural Genomics Institute at Shenzhen, Chinese Academy of Agricultural Sciences, Shenzhen 518120, China

Dear Editor,

For any species, a high-quality reference genome is the basis for almost all kinds of genomic analysis [1]. However, for decades the reference sequences of important eukaryotic genomes were incomplete due to the missing repetitive genomic regions including both tandem repeats such as centromere, telomere, and ribosomal DNA, and interspersed repeats like transposons and segmental duplications. The incomplete reference genomes not only cause data analytical mistakes like false-positive variant calls but also impede the studies of repeat-related diseases such as cancer and infertility [2, 3]. Fortunately, the generation of Pacbio high-fidelity (HiFi) long sequences and Oxford Nanopore Technology (ONT) ultra-long (UL) sequences provides the opportunity of solving repeat assembly problems because of their advantages in accuracy and length, respectively, and a list of complete (T2T, or Telomere to Telomere) or near-complete reference genomes of important eukaryotic species like human [4], *Arabidopsis* [5], rice [6] and tomato [7] have been built recently.

Because there has been no assembler that can generate complete reference genomes purely automatically until now, these T2T projects all need large amounts of manual curations. These manual works focus on generating continuous and correct sequences in repetitive regions in some steps of genome assembly such as contig assembly, scaffolding, polishing and gap filling. However, due to the high similarity between the repeat copies, the sequences from different copies may be aligned by mistake, which leads to mis-assemblies in these steps. To filter these false-positive alignments and ensure the correctness of repeat assembly, copy-specific features like Single Nucleotide Polymorphisms (SNPs) and structural variations have to be used. More specifically, the alignments in which the copy-specific features from two sequences do not match should be identified as false-positive. Due to the difficulty and high computational cost of SNP and Structural Variation (SV) detection, in practice, people use rare *k*-mers (subsequences of *k* nucleotides appearing in the whole genome for only a small number of times) as markers to replace these copy-specific features, and the alignments with rare *k*-mers mismatching should be removed. This strategy has been widely used by T2T-related automatic tools like CentroFlye [8], Abruijn [9], and manual human T2T assembly. However, due to the lack of a visualization tool that can show the match of rare *k*-mers in alignments (the existing alignment visualization tools such as IGV [10] show the alignments without *k*-mer matching), it is extremely tedious and time consuming to carry out this strategy manually. This is the reason that other T2T or near-T2T projects choose not to or use a rougher strategy to filter false-positive alignments, leading to lower correctness than human T2T assembly. Here we have developed an efficient alignment visualization tool called RAviz to meet this need. With the alignments and corresponding *k*-mer matching profiles clearly visualized by RAviz, it is much easier and time-efficient for the users to decide which are the false alignments and remove them in T2T assembly projects.

**Figure 1:**
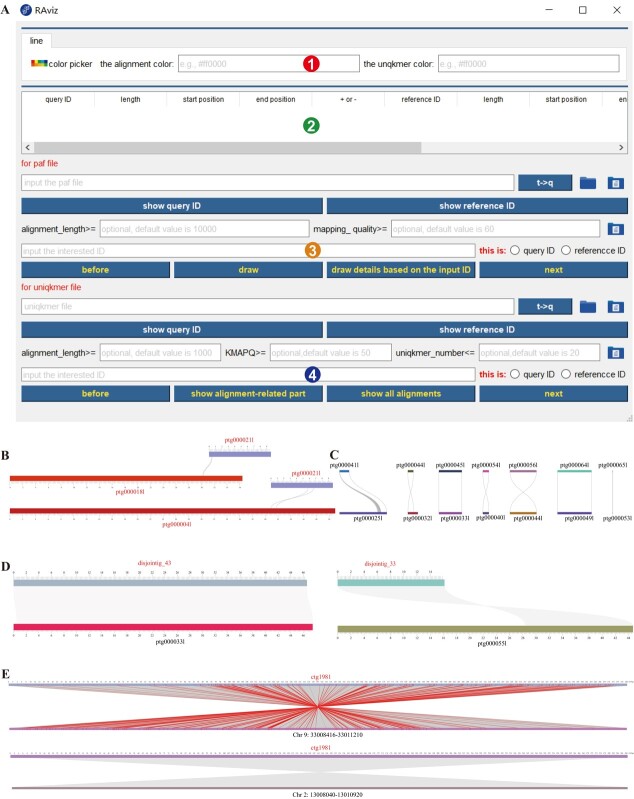
The interface and examples of RAviz. **(A**) The interface of RAviz: (i) parameter module in which the parameters for drawing are inputted; (ii) data module which shows the original data related to drawing in the form of a table; (iii) PAF drawing module for specifying the input PAF file and related parameters; and (iv) Rare *k*-mer drawing module for specifying the self-defined input file from a pre-processing program and related parameters. (**B**) An example showing all alignments related to a specified sequence. (**C**) An example showing alignments globally. (**D**) Two examples of visualization with only alignment areas (shown in grey). The two sequences in left alignment are from the same strand of genome while those in right alignment are from different strands. (**E**) An example that explains how RAviz helps detect false-positive alignments in repetitive regions. The two alignments are also two examples of visualization with both alignment area and rare *k*-mer matching profile (each pair of matching rare *k*-mers is shown as a red line between them). In both of the two alignments, the two sequences are from different strands.

RAviz is a Windows- or MacOS-based open-source software that is able to visualize the alignments between any types of DNA sequences such as reads, contigs, scaffolds or reference genomes in three different modes (Fig. 1A). First, in the global mode, the alignments in the whole genome are drawn in one or more continuous pages to show the users an overall situation (Fig. 1C). Second, two sequences can be specified by IDs and the alignments between them are shown. Third, one sequence is specified and all the alignments related to it are shown (Fig. 1B). In each mode, the users can filter the shown alignments by two types of mapping scores: MAPQ (a popular mapping score for evaluating the quality and confidence of each alignment) and KMAPQ (a mapping score defined by RAviz according to the matching of rare *k*-mers for the same purpose). If a specific region of an alignment needs to be visualized, RAviz allows the users to zoom in by pressing the ‘Ctrl’ key and operating the mouse scrollers, and to move by dragging with mouse.

RAviz allows two types of input. The standard PAF (a type of format of sequence alignment, which can be generated from minimap2 [11]) files can be directly input if the users only need to visualize the alignment areas without showing the *k*-mers (Fig. 1D). Otherwise, a pre-processing program (https://github.com/xianjia10/kmer-map.git) of RAviz needs to be run for generating a self-defined input file containing all necessary information such as alignment areas, *k*-mer positions and *k*-mer matching from PAF files and FASTA (a type of format of DNA sequences) files, and then both alignment area and the matching of rare *k*-mers can be shown for each alignment with this input file (Fig. 1E). The pre-processing program needs to be run on a Linux system, as the alignment and *k*-mer files are comparatively large files in most situations.

Fig. 1E shows a real example in which RAviz helps detect false-positive alignments in repetitive regions. In this example, a contig from tomato genome ctg1981 was aligned simultaneously to the two copies (Chr9:33008416-33 011 210 and Chr2:13008040-13 010 920) of an interspersed repeat on tomato reference genome by minimap2. Due to the high mapping scores (MAPQ = 60) of the two alignments, it is difficult to decide which copy is correct for T2T assembly. However, with the rare *k*-mer matching profile visualized by RAviz, it is easy to see that the alignment to chromosome 9 is supported by a large amount of rare *k*-mers while the alignment to chromosome 2 is a typical false-positive and should be removed.

RAviz was implemented by python 3.8 and PyQt5. In the implementation, the time-efficiency and memory-efficiency of the software have to be guaranteed considering the huge amounts of alignments and rare *k*-mers existing for almost any eukaryotic genome. For example, tomato genome (a homozygous diploid genome with ~775 M nucleotides) contains about 10.9 G unique *k*-mers (*k*-mers appearing in the whole genome only once) and there are 70 871 alignments between the corresponding HiFi contigs (not to mention reads) and reference genome. To improve the memory efficiency, the pre-processing program of RAviz stores *k*-mer positions instead of *k*-mer sequences. To improve the time-efficiency, instead of sequential searching in the input file, RAviz builds two index files that contain the mapping from reference and query IDs to the corresponding line numbers in the file, so that the related alignment information can be found ultrafast by a given reference or query. For generating an input file of 900 Mb, RAviz uses 0.7 hours with 18 CPUs.

To conclude, RAviz can efficiently visualize the sequence alignments in repetitive genomic regions with a rare *k*-mer matching profile, and thus is able to help the users detect and remove false positive alignments and generate high-quality assembly in T2T reference genome building projects. In the future, we will add the interactive functions in the next version of RAviz and develop the Linux version.

## Acknowledgements

This work was supported by National Natural Science Foundation of China (Grant No. 32100501) and Shenzhen Science and Technology Program (Grant No. RCBS20210609103819020).

## Author contributions

W.P., Y.Y., and D.X. designed this study. D.X., Y.Y., Y.S., X.Z, and D.G developed RAviz and analysed data. D.X., Y.Y., and W.P. wrote the paper.

## Data availability

The RAviz and testing data can be freely downloaded at https://github.com/xianjia10/RAviz.git. The manual and videos can be found in the tutorial subfolder under the installation directory of RAviz.

## Conflict of interest

The authors have declared no competing interests.
